# A dexterous fluorescence sensor for diversified-biomarker detection using cyclic EDC–CHA (EDCHA) system

**DOI:** 10.1007/s44211-025-00791-z

**Published:** 2025-06-25

**Authors:** Jie Du, Jinjuan Liu, Hongyan Li

**Affiliations:** Department of Health Examination Center, Shaanxi Provincial People Hospital, Xi’an, China

**Keywords:** Fluorescent assays, Cascade isothermal amplification, MicroRNAs detection, Protein detection

## Abstract

Monitoring the levels of various biomarkers, such as nucleic acids and proteins, is crucial for early disease diagnosis, evaluating treatment efficacy, and predicting patient prognosis, thereby aiding clinical decision-making. Herein_**,**_ a robust double-circuit fluorescence assay combining the advantages of EDC and CHA called EDCHA is proposed. The EDCHA includes two cycles, EDC system identifies the target miRNA, realizes first signal amplification and releases an initiator chain to trigger the CHA system. CHA system realizes second signal amplification by opened hairpin structures and different fluorophore groups. In addition, by replacing the waste chain in the EDC system with fluorescent-labeled aptamer, the EDCHA can simultaneously detect both miRNA and protein. The EDCHA was verified with the specific detection of miRNA-21 with a low detection limit (LOD) of 77.18 pM. Furthermore, our method enables the simultaneous detection of two types of biomarkers—nucleic acids and proteins—with a low detection limit (LOD) of 0.94 nM for the AD-related protein AβO. These results demonstrate the enormous potential of EDCHA in diverse biomarkers analysis and cancer diagnosis.

**Graphical abstract:**

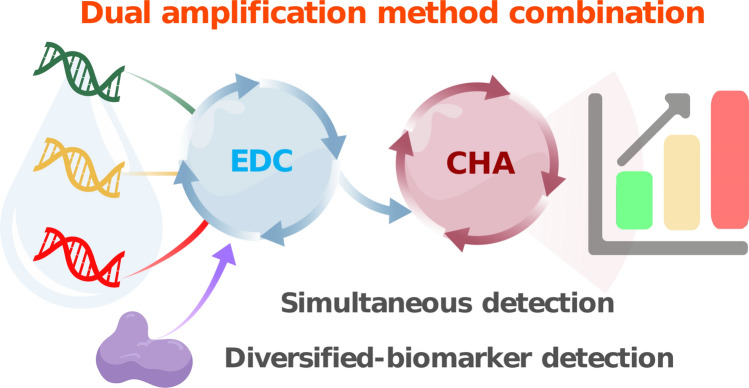

**Electronic supplementary material:**

The online version of this article (10.1007/s44211-025-00791-z) contains supplementary material, which is available to authorized users.

## Introduction

The progression of many diseases is closely related to the levels of various biomarkers, such as nucleic acids [1] and proteins [2]. Monitoring multiple biomarkers enables early disease diagnosis, evaluation of treatment efficacy, and prediction of patient prognosis, thus providing critical information for clinical decision-making [3]. MicroRNAs (miRNAs) are a class of non-coding single-stranded RNA molecules encoded by endogenous genes of approximately 19–24 nucleotides in length, which are involved in post-transcriptional gene expression regulation in plants and animals [4]. These small RNAs are actively involved in a variety of cellular physiological processes, such as cell development, differentiation, proliferation, and apoptosis [5]. Recent studies have demonstrated that abnormal expression of miRNAs is related to many diseases [6]. Thus, portable and sensitive detection of multiple miRNA targets is urgently needed for early diagnosis of these diseases.

Due to the relatively low expression levels of miRNAs in living cells, various signal amplification techniques have been devised to enable sensitive and precise miRNA detection. Among them, quantitative reverse transcription polymerase chain reaction (qRT-PCR) stands as the gold standard for detecting miRNAs in clinical samples [7]. However, it demands complex procedures and costly equipment to meticulously control reaction temperatures. Thus, many isothermal amplification strategies, such as nuclease-assisted amplification, exponential isothermal amplification (EXPAR) [8], loop-mediated isothermal amplification (LAMP) [9], strand displacement amplification (SDA) [10], and rolling circle amplification (RCA) [11], have been proposed to avoid thermocycling. However, these methods depend on enzyme-mediated reactions to detect miRNA, and these reactions are sensitive to environmental conditions, which prevent their wide applications.

Therefore, many enzyme-free signal amplification strategies have been developed, including hybridization chain reaction (HCR) [12], catalyzed hairpin assembly (CHA) [13] and entropy-driven circuit (EDC) [14]. Among them, the HCR and CHA are driven by free energy change of base pair formation. However, hairpin structures involved in these reactions can lead to complex probe design and the generation of false positive signals. Unlike the HCR and CHA, EDC is based on entropy increase in the reaction system to detect miRNA, in which toehold-mediated chain displacement ensured target regeneration through the catalytic circuit. The simplified probe design and reduced reversibility at each step render EDC a more promising approach for miRNA detection. Recently, researchers have combined EDC with other isothermal amplification strategies to detect miRNA with higher sensitivity [15]. However, these methods are rarely applied to multiple miRNA detections.

The method of protein detection based on aptamers is referred to as aptamer-affinity detection technology [16]. This approach leverages the high affinity and specificity of aptamers for target proteins, serving as a valuable detection tool extensively applied in fundamental research, clinical diagnostics [17], and pharmaceutical development. Compared to traditional antigen–antibody recognition methods, such as enzyme-linked immunosorbent assay (ELISA) or Western blot (WB), aptamer-based protein detection provides benefits including high chemical stability, simple synthesis and modification, and cost-effectiveness, showcasing substantial application potential and opportunities. AβO protein, a critical biomarker for Alzheimer’s disease (AD), is generated through the proteolytic processing of amyloid precursor protein (APP) by β- and γ-secretases, leading to the formation of Aβ40 (non-amyloidogenic) and Aβ42 (amyloidogenic) peptides [18]. The accumulation of AβO contributes to synaptic dysfunction, neuronal toxicity, and neuroinflammation hallmarks of AD pathology [19]. In parallel, miRNA-125, another key biomarker, is upregulated in AD brain tissue and cerebrospinal fluid (CSF), promoting neuroinflammation and neuronal dysfunction by downregulating NR3C2 and Bcl-W. It also modulates the ERK/MAPK pathway, which is linked to Aβ-induced neurotoxicity [20–22]. In addition, AβO interacts with neuronal receptors like NMDAR and EphB2, leading to synaptic impairment [23, 24]. Given their roles in AD pathology, the simultaneous detection of miRNA-125 and AβO could provide a more comprehensive biomarker panel for improved disease diagnosis and monitoring. Currently, methods capable of simultaneously detecting two types of biomarkers (nucleic acids and proteins) are rare. However, multiplex detection methods can provide more comprehensive biological information, enhancing the accuracy and sensitivity of disease diagnosis.

In this study, combined with the advantages of EDC and CHA, we developed a double-circuit-assisted fluorescence biosensor for diversified-biomarker detection. The double-circuit reaction contained two parts: (1) target identification and first signal amplification step based on the EDC system; (2) second signal amplification assisted by CHA and signal generation realized by opened hairpin structures and different fluorophore groups. This biosensor could detect multiple cancer-related miRNAs simultaneously and detect AD-related miRNA and protein at the same time aided by AβO specific aptamer. Moreover, we have successfully applied this double-circuit biosensor to miRNAs extracted from cell lines, and the test results are consistent with previous studies. This biosensor platform could be tailored to detect other disease-related miRNAs smoothly with little change in sequence design, showing promising potential for early diagnosis of various diseases.

## Experimental section

### Reagents

All nucleic acids were synthesized and purified by Sangon Biotech (Shanghai, China), and the detailed sequences are listed in Table 1. The DNA marker, Sybe gold, fetal bovine serum (FBS), and penicillin–streptomycin were purchased from Vazyme (Jiangsu, China). Dulbecco’s modified Eagle medium (DMEM) was purchased from Cytiva (Shanghai, China). Tris(hydroxymethyl)-aminomethane hydrochloride (Tris–HCl), magnesium chloride hexahydrate (MgCl_2_·6H_2_O), and potassium chloride (KCl) were purchased from Beyotime (Shanghai, China). AβO was purchased from Peptide Institute Inc. (Japan).

**Table 1:** The sequences and modification of DNA used in this work

Name	Sequences (5′ to 3′)
miR-21	UAGCUUAUCAGACUGAUGUUGA
S-1	CTGAGGATGTCACACGCACAGTGCTGAATACCTCAACATCAGTCTGATAAGCTA
S-1-BHQ2	CTGAGGATGTCACACGCACAGTGC/iBHQ2dT/GAATACCTCAACATCAGTCTGATAAGCTA
W	CCTCAGCACTGTGCGTGTGACATCCTCAGCA
I-21	TCAGACTGATGTTGAGGTATGGTCGATAAT
I-21-Cy5	TCAGACTGATGTTGAGGTATGGTCGATAAT-Cy5
H1-21	FAM-GTCGATAATTATGGGTATGATTATCGACCATACC-BHQ1
H2-21	ATGGGTATGGTCGATAATCATACCCATAATTAT
F-1	TCAGACTGATGTTGAGGTATTCAGCACTGTGCGTGTGACATCCTCAG
miR-155	UUAAUGCUAAUCGUGAUAGGGGU
S-2	CTGAGGATGTCACACGCACAGTGCTGAGATGCACCCCTATCACGATTAGCATTAA
I-155	AATCGTGATAGGGGTGCATCGGTCGATAAT
H1-155	TR-GTCGATAATTATGGCATCGATTATCGACCGATGC-BHQ2
H2-155	ATGGCATCGGTCGATAATCGATGCCATAATTAT
F-2	AATCGTGATAGGGGTGCATCTCAGCACTGTGCGTGTGACATCCTCAG
miR-125	UCCCUGAGACCCUAACUUGUGA
S-3	CTGAGGATGTCACACGCACAGTGCTGAAGTCTTCACAAGTTAGGGTCTCAGGGA
I-125	GACCCTAACTTGTGAAGACTGGTCGATAAT
H1-125	Cy5-GTCGATAATTATGAGACTGATTATCGACCAGTCT-BHQ3
H2-125	ATGAGACTGGTCGATAATCAGTCTCATAATTAT
F-3	GACCCTAACTTGTGAAGACTTCAGCACTGTGCGTGTGACATCCTCAG
I-125’	CACCCTAACTTGTGATGGGGTGTCGATAAT
H1-125’	FAM-GTCGATAATTATGTGTACCATTATCGACACCCCA-BHQ1
H2-125’	TATGTGTACCTGTCGATAATGGTACACATAATTAT
S’	CGCACCCGCCCCAACACCACAGGCACCCCATCACAAGTTAGGGTCTCAGGGA
F’	CACCCTAACTTGTGATGGGGTGCCTGTGGTGTTGGGGCG
A1	Cy5-ACGCCTGTGGTGTTGGGGCGGGTGCGGT-BHQ3
A2	Cy5-CAACGCCTGTGGTGTTGGGGCGGGTGCGGTTG-BHQ3
A3	Cy5-ACCAACGCCTGTGGTGTTGGGGCGGGTGCGGTTGGT-BHQ3
A4	Cy5-TGACCAACGCCTGTGGTGTTGGGGCGGGTGCGGTTGGTCA-BHQ3
A5	Cy5-ACTGACCAACGCCTGTGGTGTTGGGGCGGGTGCGGTTGGTCAGT-BHQ3

FAM: 6-carboxyfluorescein, TR: Texas Red, Cy5: Cyanine 5

### Instruments

The fluorescence intensity was measured by the Thermo Fisher Microplate reader (Multiskan SkyHigh). The fluorescent images and gel images were recorded by the Bio-Rad fluorescence imaging analysis system (ChemiDoc MP).

### Detection of microRNA

Ternary duplex (I/S/W), H1, and H2 were heated to 90 °C and then gradually cooled to room temperature, respectively. Before use, the ternary duplex was purified using a LifeFeng Small Fragment DNA Mini Purification Kit (DK414) to remove most of the unhybridized single strands. The purified ternary duplex was quantified by polyacrylamide gel electrophoresis (PAGE) using the unpurified ternary duplex as a reference, yielding a recovery rate of approximately 70%. The purified ternary duplex was then stored as a stock solution in the refrigerator. In a typical assay, a reaction mixture contained target (T) of varying concentrations, 20 nM ternary duplex, 50 nM fuel (F), 500 nM H1, and 500 nM H2 in 50 mM Tris–HCl (pH = 8) buffer containing 50 mM KCl and 5 mM MgCl_2_. The mixture was incubated at 25 °C for 3 h and then allowed to transfer to a 96-well microplate to measure the fluorescence signal. F and F_0_ refer to the fluorescence intensity before and after adding the target, respectively.

### Detection of miRNA-125 and AβO

Ternary duplex (A/I-125′/S′), H1-125′, and H2-125′ were heated to 90 °C and then gradually cooled to room temperature before use, respectively. The ternary duplex was also subjected to the afore-mentioned purification steps before use, and the resulting stock solution was stored in the refrigerator. To detect AβO, different concentrations of AβO and different concentrations of miRNA-125 were added to the reaction mixture containing 50 nM ternary duplex, 70 nM fuel (F′), 500 nM H1-125′, and 500 nM H2-125′ in 50 mM Tris–HCl (pH = 8) and 10 mM MgCl_2_ buffer. Then the mixture was incubated at 25℃ for 3 h and transferred to measure the fluorescence intensity.

### Native polypropylene gel analysis

A 15% native polyacrylamide gel was prepared and polymerized for 25 min at room temperature. Then the gel was transferred to the electrophoresis chamber and soaked in 1 × TBE buffer. After adding samples to the loading well, PAGE was run at 120 V constant voltage for 30 min. After staining in a newly prepared Sybe gold solution (15 min), the gel was characterized using the ChemiDoc MP imaging system. A 20 bp DNA ladder was loaded alongside the samples to serve as a molecular size standard in the PAGE analysis.

### Cell culture and RNA extraction

HeLa cells, MCF-7 cells, HEK-293 cells, and MCF-10A cells were cultured in Dulbecco’s modified Eagle’s medium (DMEM) containing 10% FBS and 1% penicillin–streptomycin. All the cells were cultured at 37℃ in a humidified atmosphere containing 5% CO_2_. When the cell growth reached 70%–80%, the cells were transferred to a centrifuge tube for RNA extraction. The RNA was obtained using a Vazyme Tissue Total RNA Isolation Kit.

## Results and discussion

### The working principle

**Scheme 1.**
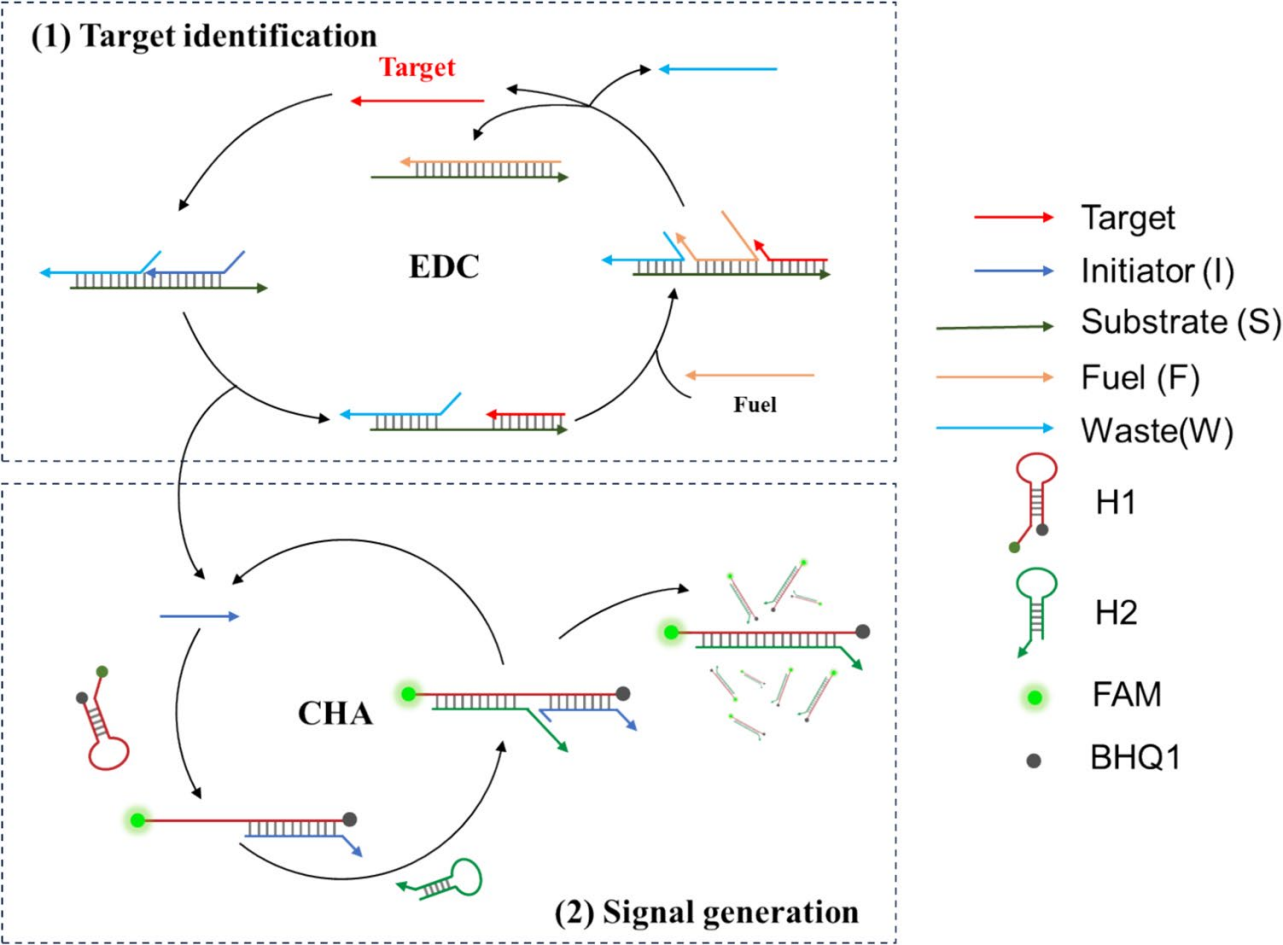
Schematic illustration of proposed cascade isothermal amplification strategy for the detection of miRNA

The methodological principle for detecting miRNA by cascade isothermal amplification is illustrated in Scheme 1. The proposed method includes two cycles: an entropy-driven catalytic (EDC) amplification reaction and a catalytic hairpin assembly (CHA) reaction. As shown in Scheme 1, the EDC circuit contains a pre-hybridized three-stranded composite substrate (I-S-W) and a fuel strand (F). When the target miRNA (T) appears, the bases exposed at the chain S in triplex serve as toehold and allow chain T to replace chain I to form an intermediate. Immediately afterward, the chain fuel (F) undergoes another strand displacement reaction. During this process, both chain T and W are replaced by chain F, realizing the recycling of the target. Meanwhile, a released chain I triggers the CHA progress, achieving cascade signal amplification. The CHA circuit consists of two RNA hairpins (H1 and H2). H1 contains the sequence which is perfectly complementary to the sequence of chain I and the loop region. H2 contains the sequence which is partially complementary to H1. H1 and H2 are metastable and cannot hybridize spontaneously, because the complementary sequences are placed in the stems. However, chain I can bind to the exposed toehold domain of H1 and unfold H1 to generate an intermediate, which can further initiate strand displacement of H2 and release chain I to initiate a new catalytic cycle. Moreover, the phosphorylated ends of H1 were labeled with a fluorophore and a quencher. The unfolding of the H1 causes its attached fluorophore to move away from the quencher, resulting in the restoration of the fluorescence signal.

### Feasibility study

**Fig. 1:**
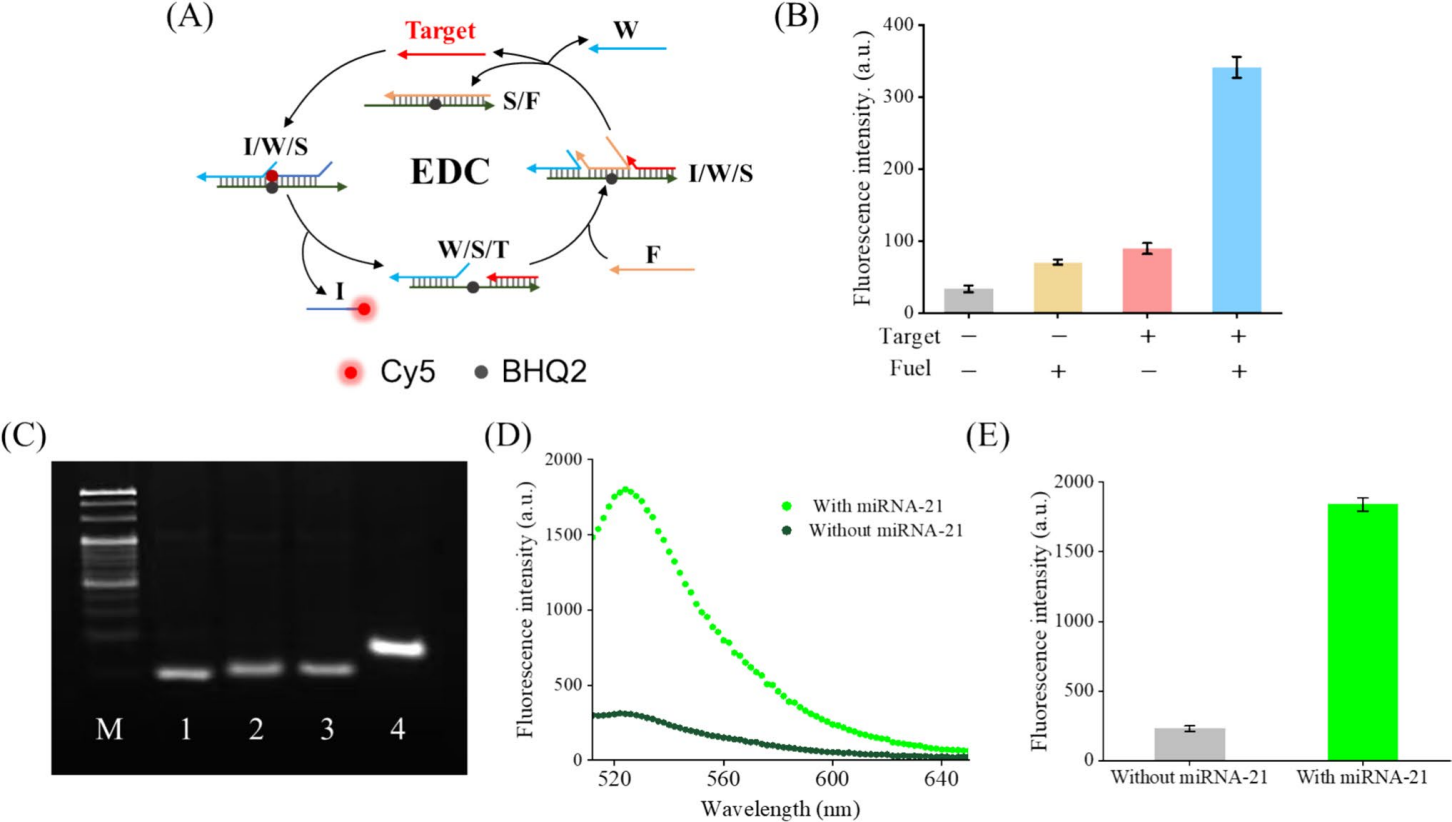
Feasibility of the proposed strategy. **A** Schematic showing the working principle of EDC assays. **B** Fluorescence intensity of EDC systems with different component combinations. Sample 1: neither target nor fuel; Sample 2: fuel only; Sample 3: target only; Sample 4: both target and fuel. Assay conditions: 20 nM ternary duplex (purified at a 1:2:3 ratio), 5 nM T (or none), and 50 nM F (or none) at 25 °C for 2 h. **C** PAGE analysis of the feasibility of CHA reaction. Lane M: 20bp Marker; Lane 1: H1; Lane 2: H2; Lane 3: H1 + H2; Lane 4: H1 + H2 + I. Assay conditions (e.g., Lane 4): 5 nM I, 500 nM H1, 500 nM H2 at 25 °C for 2 h. **D** Fluorescence emission spectra of the EDC–CHA system with or without miRNA-21. Assay conditions: 20 nM ternary duplex (purified at a 1:2:3 ratio), 1 nM T, 70 nM F, 500 nM H1, 500 nM H2 at 25 °C for 3 h. Fluorophore: FAM. **E** Fluorescence intensity of the EDC-CHA system with or without miRNA-21. Assay conditions were identical to those in panel **D**. Error bars, SD, *n* = 3

Oncogenic miRNA-21 was selected as a model target in this part. It has been reported that aberrant miRNA-21 expression is closely linked to various types of tumors. First, we labeled a fluorophore Cy5 and a quencher BHQ2 to chain I and chain S, respectively, to test the feasibility of the EDC reaction (Fig. 1A). When the strand displacement reaction of chain I occur, Cy5 will move away from BHQ2 and result in the generation of the fluorescence signal. As shown in Fig. 1B, almost no fluorescent signal could be observed at sample 1 and 2 which did not contain miRNA-21. When miRNA-21 was added, a weak fluorescent signal was observed at sample 3. Only when both miRNA-21 and chain fuel were added, the sample could generate bright fluorescent signal. These results indicated that the strand displacement reaction of chain I was initiated by the target miRNA and chain F realized the recycling of the target miRNA. The fluorescence gain after EDC signal amplification was about 4.8-fold. In addition, to prove that in the presence of chain I and hairpin structure H1 and H2 which indeed undergo CHA, a PAGE was carried out. As shown in Fig. 1C, bands of H1 and H2 conjugates appeared at lane 4, but the same phenomenon is not observed at other lanes. Moreover, increasing the amount of the I strand results in a more complete reaction (Fig. S1). These phenomena indicate that chain I can trigger H1 and H2 to undergo CHA. Moreover, the feasibility of the proposed strategy was further examined by fluorescence spectra. The phosphorylated ends of the H1 label with the fluorophore FAM and the quencher BHQ1. As shown in Fig. 1D and E, only a low fluorescence intensity was detected in the spectrum of the sample without miRNA-21. However, relatively high fluorescence intensity was observed in the spectrum of samples with 1 nM miRNA-21. The fluorescence gain after EDCHA dual signal amplification was about 8.0-fold, and the fluorescence peak was at 518 nm.

### Optimization of reaction conditions

**Fig. 2:**
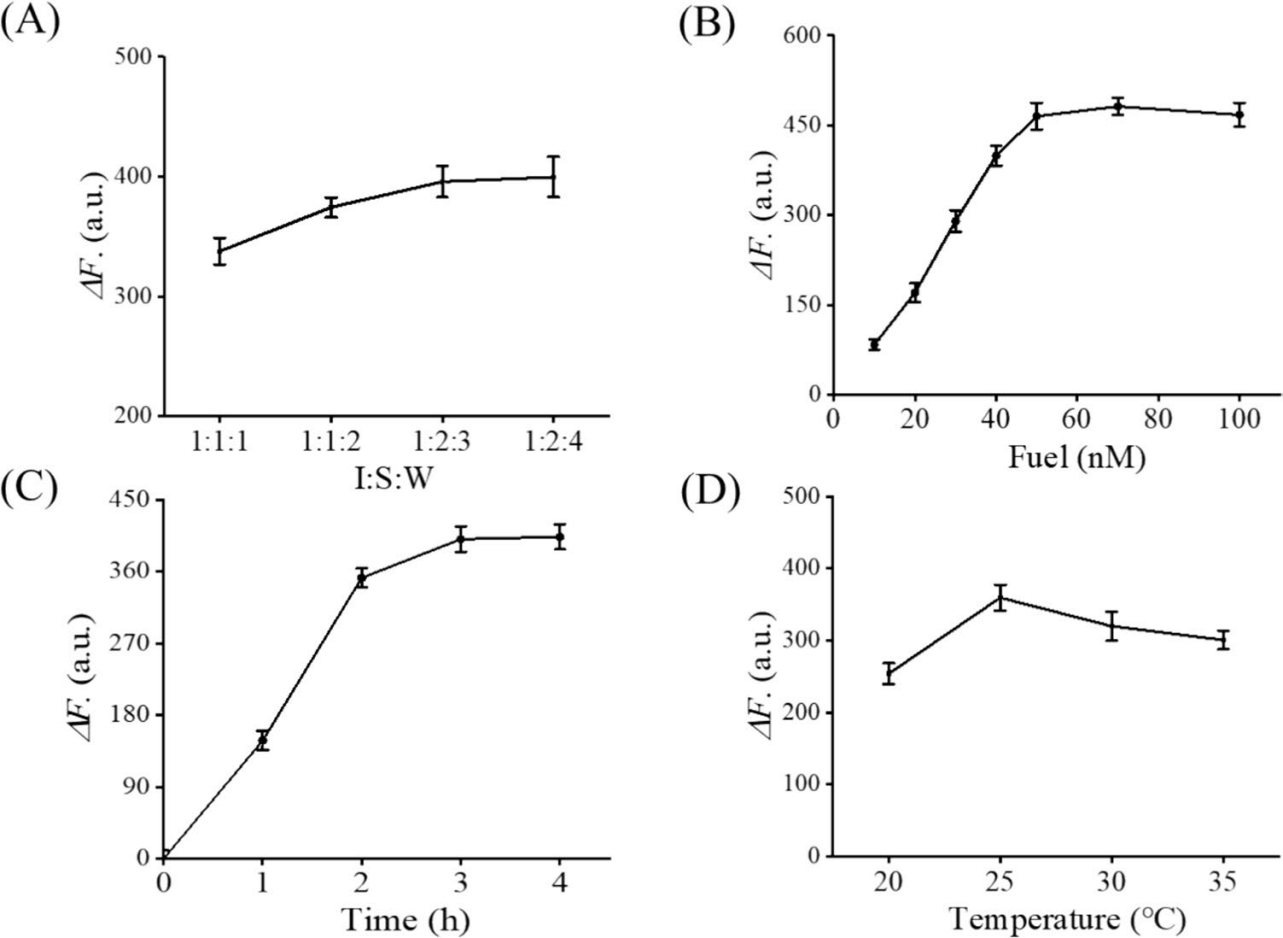
Optimization of experimental parameters. **A** Ratio of chain I, S, and W. Assay conditions: 20 nM ternary duplex (purified at a 1:2:3 ratio), 1 nM T, 70 nM F, 500 nM H1, 500 nM H2 at 25 °C for 3 h. **B** Concentration of chain F. Assay conditions: 20 nM ternary duplex (purified at a 1:2:3 ratio), 1 nM T, varying concentrations of F, 500 nM H1, 500 nM H2 at 25 °C for 3 h. **C** Reaction time. Assay conditions: 20 nM ternary duplex (purified at a 1:2:3 ratio), 1 nM T, 70 nM F, 500 nM H1, 500 nM H2 at 25 °C for different durations. **D** Reaction temperature. Assay conditions: 20 nM ternary duplex (purified at a 1:2:3 ratio), 1 nM T, 70 nM F, 500 nM H1, 500 nM H2 for 3 h at various temperatures. Error bars, SD, *n* = 3

To boost the detection performance, several reaction conditions, such as the ratio of chain S, I and W (I/S/W), the concentration of chain F, and the reaction time and temperature, were optimized. The difference value of fluorescence intensity between the sample in the presence of miRNA-21 and the sample in the absence of miRNA-21 (ΔF) was used to evaluate the performance of the EDC-CHA system. As shown in Fig. 2, ΔF increased with the I:S:W, concentration of chain F and the reaction time, and reached the maximum value at 1:2:3, 50 nM, and 3 h, respectively. In addition, ΔF reached the maximum value at 25 °C. Further increase in reaction temperature led to a decrease of ΔF. The increase in temperature makes the hybridization between chains less tight, resulting in an increase in background signal and a decrease in signal gain. The optimized experimental conditions were used in the subsequent experiments.

### Sensitivity of the sensing system

**Fig. 3:**
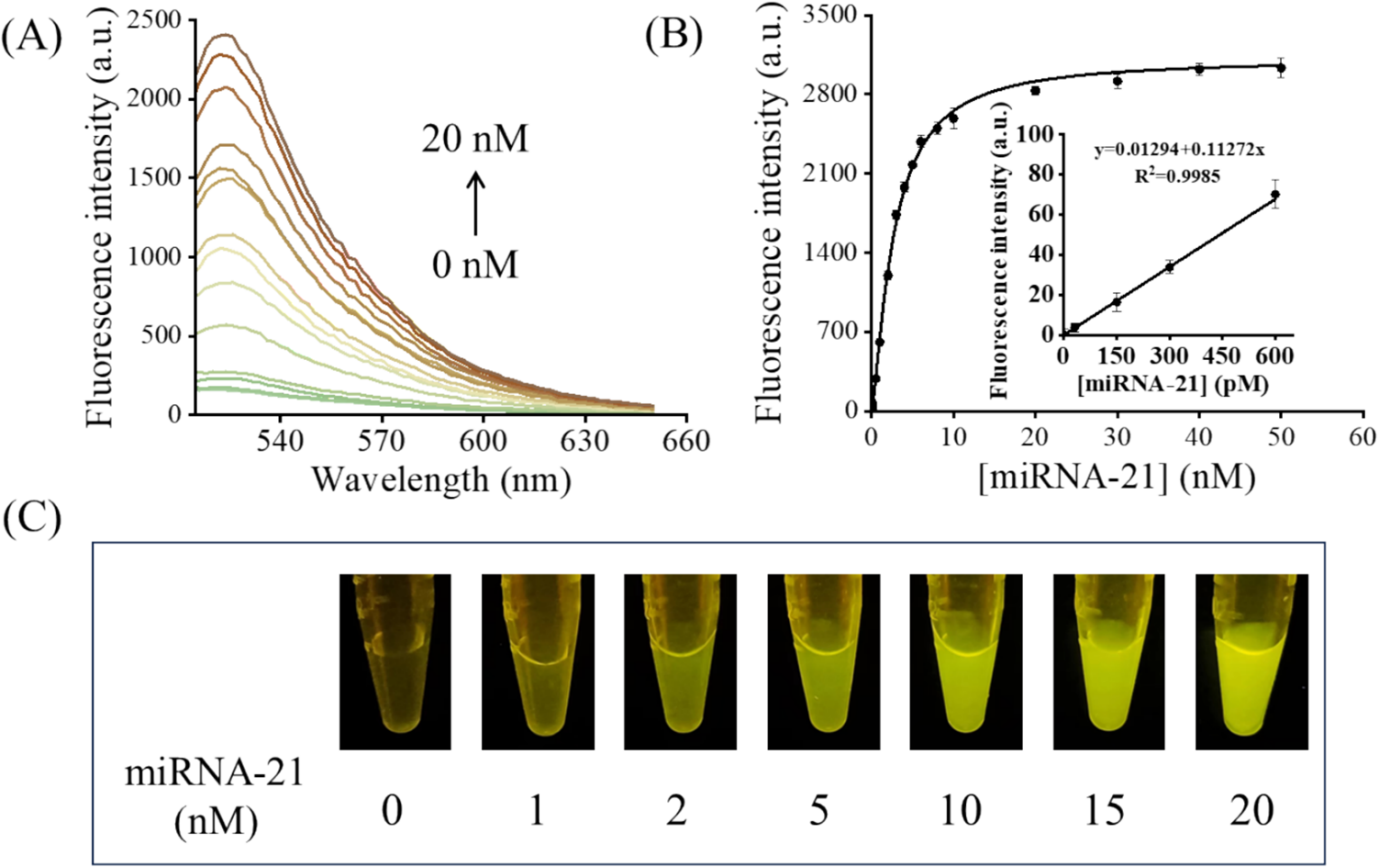
**A** Fluorescence emission spectra of the EDC–CHA system with different miRNA-21 concentrations (from 0 to 20 nM). **B** Relationship between fluorescence intensity and miRNA-21 concentration, the inset shows a linear relationship between the fluorescence intensity and miRNA-21 concentration in the range of 0 to 600 pM, in the EDC–CHA system. Error bars, SD, *n* = 3. **C** Photographs of the colorimetric determination of miRNA-21 at different concentrations. Assay conditions: varying concentrations of miRNA-21, 20 nM ternary duplex (purified at a 1:2:3 ratio), 50 nM F, 500 nM H1, 500 nM H2 at 25 °C for 3 h

Detection of miRNA by EDC or CHA alone faces the drawback of limited detection limits. Cascade isothermal amplification can enhance detection sensitivity and accuracy for miRNA identification. As shown in Fig. 3A, with the addition of miRNA-21 increasing from 0 to 20 nM, the fluorescence intensity at 520 nm gradually increased, indicating that the EDC–CHA system is effective for the detection of miRNA-21. Figure 3B shows the relationship between the fluorescence intensity of the detecting system and the concentration of miRNA-21. After fitting, a linear relationship of the fluorescence intensity for the miRNA-21 concentration range from 0 to 600 pM (*R*^2^ = 0.9985) was obtained. The detection limit was calculated to be 77.182 pM. We also conducted experiments to assess the detection limits for miRNA-155 and miRNA-125. The results, presented in Fig. S2, revealed detection limits of 156.134 pM for miRNA-155 and 108.381 pM for miRNA-125. These findings, together with the target-induced fluorescent color change shown in Fig. 3C, where the fluorescence of the reaction solution progressively intensified with increasing miRNA-21 concentration, highlight the broad applicability and robustness of our method.

### Detection of multiple miRNAs

**Fig. 4:**
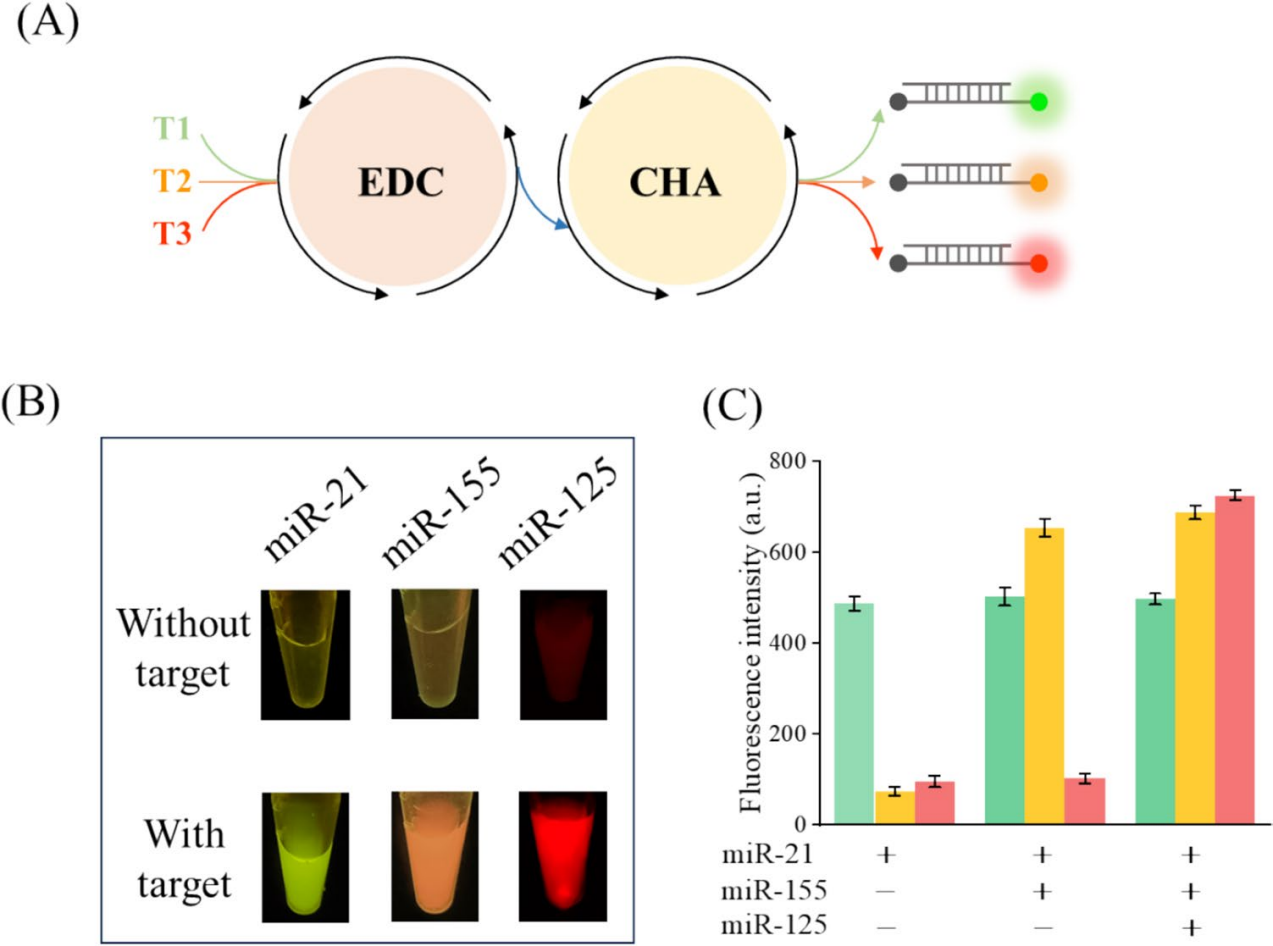
Simultaneous detection of multiple miRNAs. **A** Schematic illustration of EDC–CHA system for the detection of multiple miRNAs. T1: miRNA-21; T2: miRNA-155; T3: miRNA-125. **B** Photographs of the colorimetric determination of the EDC–CHA system with different targets. **C** Fluorescence intensity of the EDC–CHA system with different combinations of miRNA targets. Sample 1 contains miRNA-21; Sample 2 contains miRNA-21and miRNA-155; Sample 3 contains miRNA-21, miRNA-155, and miRNA-125. Assay conditions: 1 nM T, 20 nM ternary duplex (purified at a 1:2:3 ratio), 50 nM F, 500 nM H1, 500 nM H2 at 25 °C for 3 h. Error bars, SD, *n* = 3

It has been reported that the analysis of a single miRNA cannot provide enough information to allow for a reliable diagnosis of the specific disease since individual miRNAs could be linked to multiple diseases. Therefore, the detection of multiple miRNAs associated with the disease was required to generate convincing evidence needed for reliable early diagnosis and monitoring of therapeutic efficacy. EDC–CHA system also can be used to detect multiple miRNAs simultaneously by introducing specific sequences and fluorescent-labeled probes corresponding to multiple target miRNAs. As shown in Fig. 4A, H1-FAM, H1-Texas Red and H1-Cy5 combined in one pot were used to detect different miRNAs in this part, and miRNA-21, miRNA-155, and miRNA-125 were selected as model targets. Three samples containing different combinations of these three miRNA targets were tested. As shown in Fig. 4B and C, the fluorescence signal corresponding to the H1 for a given miRNA could only be obtained when the corresponding miRNA was added to the reaction. When two or three miRNAs were added simultaneously, two or three corresponding fluorescence signals were predictably observed, respectively. These results, along with the findings in Fig. S3 demonstrating that a single miRNA or the simultaneous presence of two miRNAs or three miRNAs could produce the corresponding fluorescence, further confirm that our experimental design has excellent specificity. This indicates that the method can accurately distinguish and respond to multiple miRNAs within a single reaction mixture.

### Simultaneous detection of miRNA and protein

**Fig. 5:**
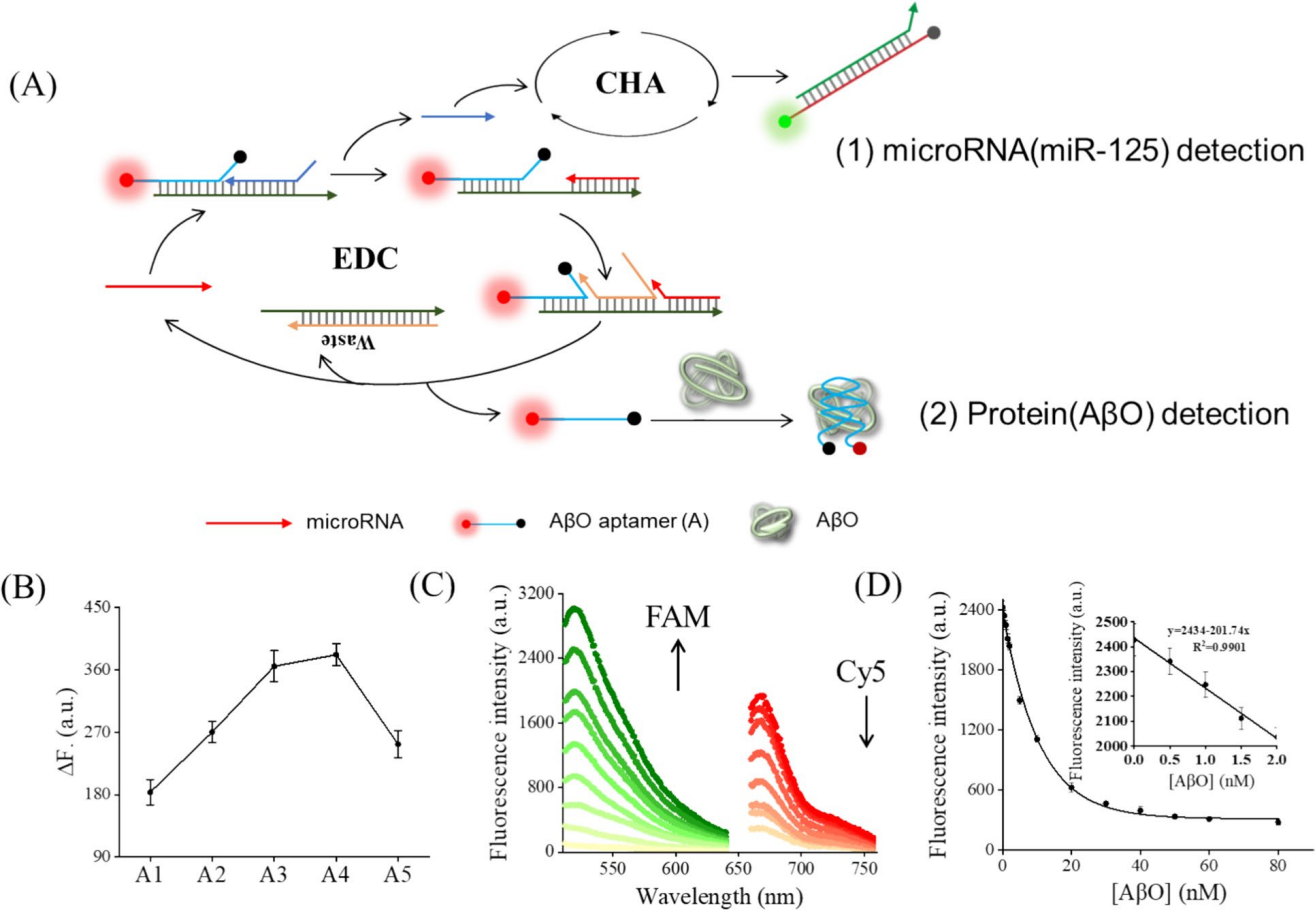
Simultaneous detection of miRNA-125 and protein AβO. **A** Schematic illustration of EDC–CHA system for simultaneous detection of miRNA-125 and protein AβO. **B** Optimization of the length of A. Assay conditions: 20 nM varying length of A, 2 nM AβO at 25 °C for 30 min. **C** Fluorescence emission spectra of the EDC–CHA system for detection of miRNA-125 and protein AβO. Assay conditions: 50 nM ternary duplex (purified at a 1:2:3 ratio), varying concentration of miRNA-125 (0–20 nM), varying concentration of AβO (0–20 nM), 70 nM F, 500 nM H1, 500 nM H2 at 25 °C for 3h. **D** Relationship between fluorescence intensity and AβO concentration, the inset shows a linear relationship between the fluorescence intensity and AβO concentration in the range of 0 to 2 nM, in the EDC–CHA system. Assay conditions were identical to those in panel **C**. Error bars, SD, *n* = 3

Sensitive and accurate multiplex detection of various sorts of biomarkers, including nucleic acid and protein, is critical for clinical disease diagnosis. Protein can be measured by different methods, among which aptamers can be used to develop aptamer-based amplification assays, based on the structure of nucleic acid. From this, we replace the chain W in the EDC circuit with fluorescent-labeled AβO aptamer (A) to detect protein AβO [25], while detecting miRNA-125. As shown in Fig. 5A, AβO aptamer (A) can be released at an EDC circuit. Released A can interact with protein AβO with high affinity and specificity. Moreover, the folding of the A causes its attached fluorophore to close to the quencher, resulting in the quenching of the fluorescence signal. To boost the detection performance, the length of A was optimized. As shown in Fig. 5B, ΔF reached the maximum value when using A4. This indicates a strong affinity between A4 and AβO, as further supported by the binding analysis in Fig. S4. Subsequently, the sensitivity of the proposed strategy was studied. As shown in Fig. 5C, with miRNA-125 and protein AβO increasing, the fluorescence intensity of FAM, which was labeled with H1-125′, gradually increased, and the fluorescence intensity of Cy5, which was labeled with A, gradually decreased, indicating that the detecting system is effective for simultaneous detection of miRNA-125 and protein AβO. In addition, as shown in Fig. 5D, the detection limit of protein AβO was calculated to be 0.94 nM.

### Detection of miRNA extracted from cell lines

**Fig. 6:**
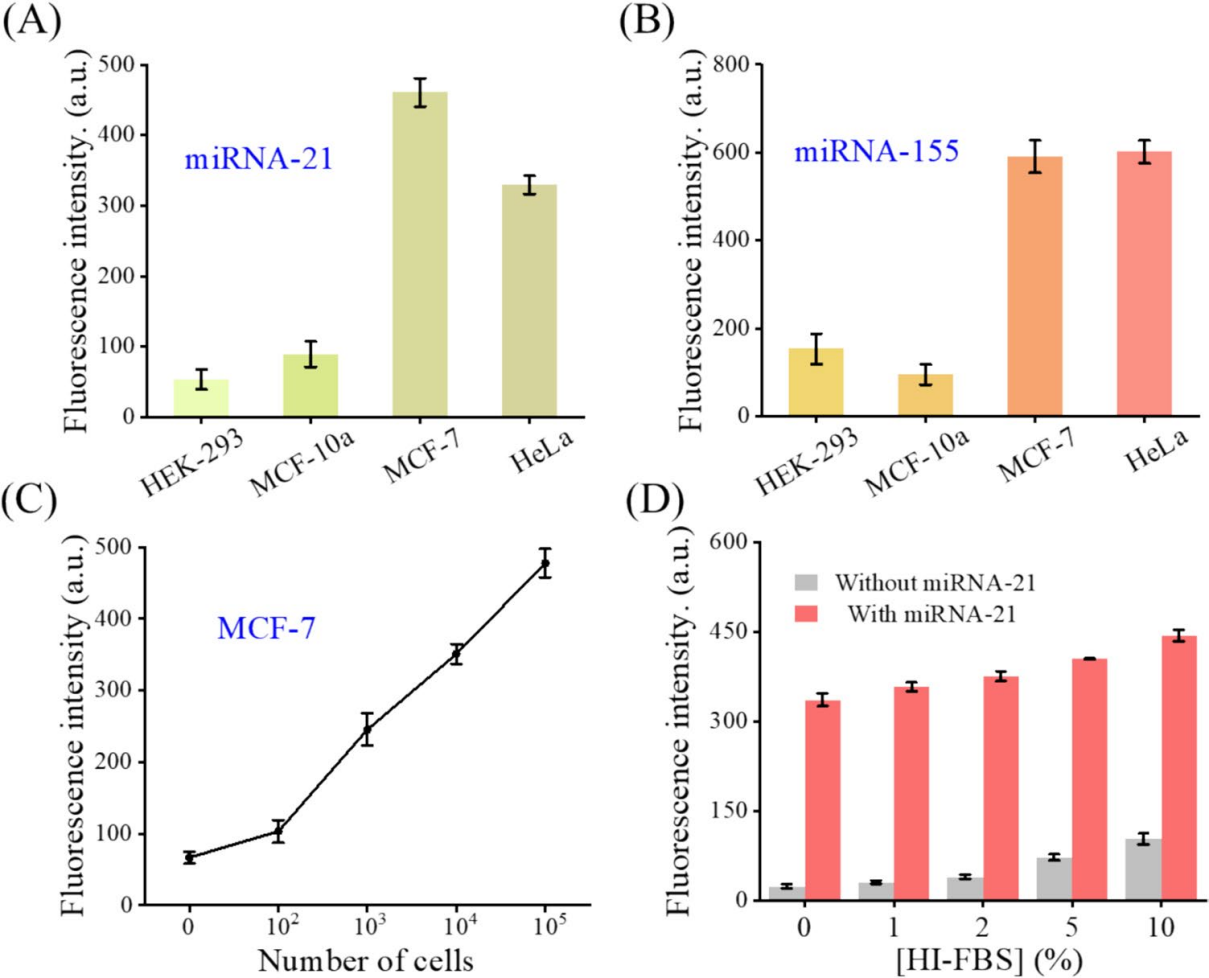
Detection of miRNA-21 (**A**) and miRNA-155 (**B**) in HEK-293, MCF-10a, MCF-7, and HeLa cell lysates. Assay conditions: 50 nM ternary duplex (purified at a 1:2:3 ratio), RNA extracted from approximately 10^5^ cells, 50 nM F, 500 nM H1, 500 nM H2 at 25 °C for 3 h. **C** Detection of miRNA-21 from different number of MCF-7 cells. Assay conditions were identical to those in panel **A**. **D** Fluorescence values and fluorescence recovery before and after adding miRNA-21 in the presence of HI-FBS at different concentrations. Assay conditions: 50 nM ternary duplex (purified at a 1:2:3 ratio), 1 nM miRNA-21, 50 nM F, 500 nM H1, 500 nM H2, varying concentration of HI-FBS at 25 °C for 3 h. Error bars, SD, *n* = 3

To demonstrate the applicability of the EDC-CHA system to complex biological samples testing, miRNA-21 and miRNA-155 extracted from different cancer cell lines were considered. As shown in Fig. 6A–C, the fluorescence intensities of the EDC-CHA system detecting different cancer cell lines and the expression levels of miRNA-21 and miRNA-155 in different cancer cell lines agreed with each other. To be specific, MCF-7 and HeLa cells were higher than HEK-293 and MCF-10a cells, which were consistent with previous research findings and further supported by the RT-qPCR results in Fig. S5. In addition, the fluorescence intensity was increased with the number of cells. Moreover, in Fig. 6D, the sensing strategy we devised has demonstrated commendable detection stability within the milieu of heat-inactivated fetal bovine serum (HI-FBS). With the increment of HI-FBS concentration, the background signal experienced an increase, while the target signal exhibited a more pronounced elevation, indicating that the fluorescence gain obtained in different environments was relatively stable. These results demonstrated that the EDC–CHA system can accurately detect miRNA in complex biological samples.

## Conclusion

In summary, we proposed a novel cascade isothermal amplification strategy based on entropy-driven catalytic (EDC) amplification reaction and catalytic hairpin assembly (CHA) reaction for the highly sensitive detection of miRNA. Compared to the EDC or CHA single-output mode, the EDC–CHA system exhibited superior amplification efficiency and increased detection sensitivity, achieving a low LOD of 77.182 pM. Moreover, the EDC–CHA system was simple and convenient to operate without expensive protein enzymes or complex thermal cycling program settings. Furthermore, the EDC–CHA system had significant potential for detecting multiple miRNAs or miRNA and protein simultaneously through rational nucleic acid sequence design, which generated convincing evidence for reliable early diagnosis and monitoring of therapeutic efficacy.

## Electronic supplementary material

Below is the link to the electronic supplementary material.

Supplementary material 1 (DOCX 396 kb)

## Data Availability

The datasets are available from the corresponding author on reasonable request.
