# Supplementary material for: A dexterous fluorescence sensor for diversified-biomarker detection using cyclic EDC–CHA (EDCHA) system
Source: Anal Sci. 2025 Jun 25;41(8):1325–34. doi: 10.1007/s44211-025-00791-z (PMC12307502; doi:10.1007/s44211-025-00791-z)
Supplement: Supplementary file 1 — Supplementary material 1 (DOCX 396 kb) [file 44211_2025_791_MOESM1_ESM.docx]

Supporting information for

A dexterous fluorescence sensor for diversified-biomarker detection using cyclic EDC-CHA (EDCHA) system

Jie Du, Jinjuan Liu*, and Hongyan Li

Department of Health Examination Center, Shaanxi Provincial People Hospital, Xi’an, China. E-mail: 15229890924@163.com

**
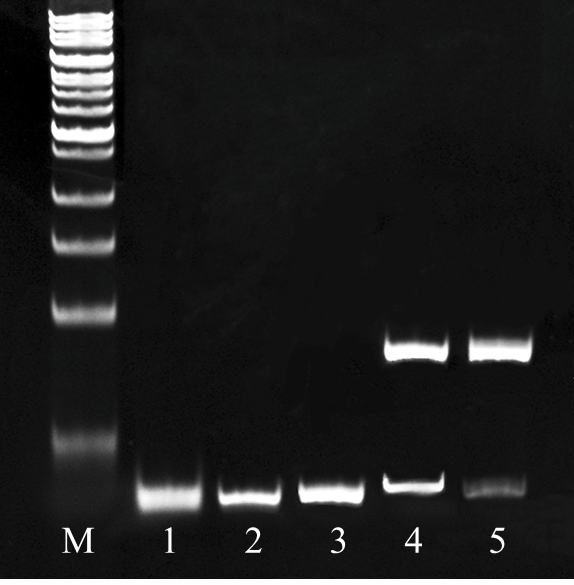
**

**Fig. S1** PAGE analysis of the feasibility of CHA reaction. Lane M: Marker; Lane 1: H1; Lane 2: H2; Lane 3: H1 + H2; Lane 4: H1 + H2 + I (5 nM); Lane 4: H1 + H2 + I (50 nM). Assay conditions (e.g., Lane 4): 5 nM I, 800 nM H1, 800 nM H2 at 25°C for 2 hours.


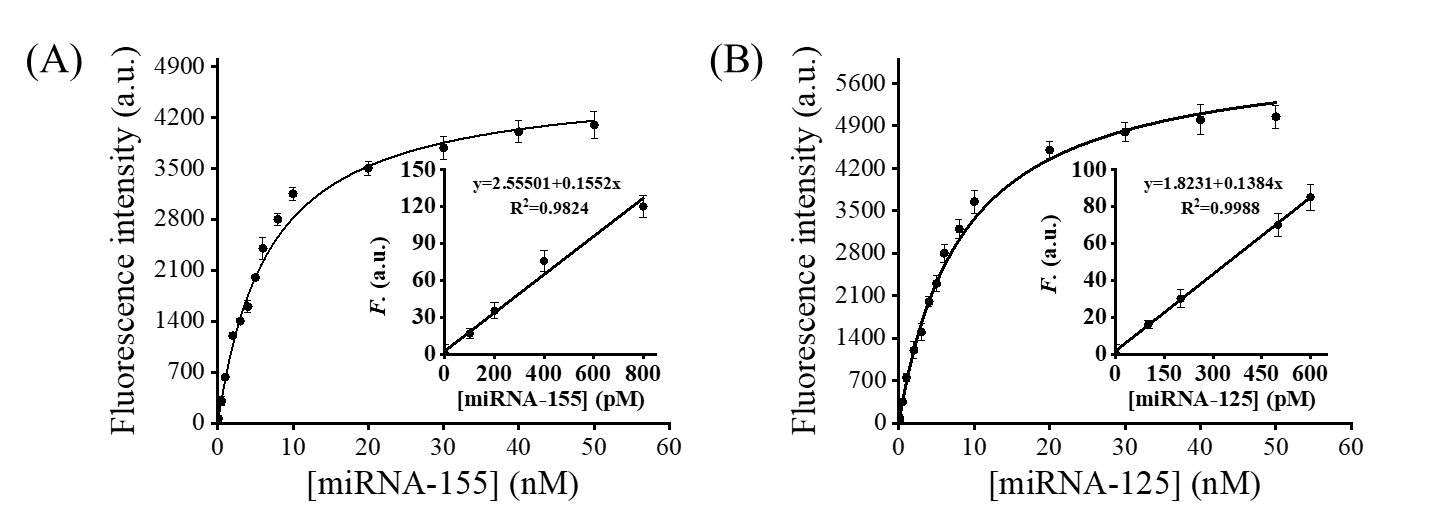


**Fig. S2** Relationship between fluorescence intensity and miRNA concentration. Panel (A) shows the relationship for miRNA-155, with an inset illustrating the linear relationship between fluorescence intensity and miRNA-155 concentration in the range of 0 to 800 pM. Panel (B) shows the relationship for miRNA-125, with an inset illustrating the linear relationship between fluorescence intensity and miRNA-125 concentration in the range of 0 to 600 pM. Assay conditions: Varying concentrations of T, 20 nM ternary duplex (purified at a 1:2:3 ratio, 50 nM F, 500 nM H1, 500 nM H2 at 25°C for 3 hours. Error bars, SD, n = 3.


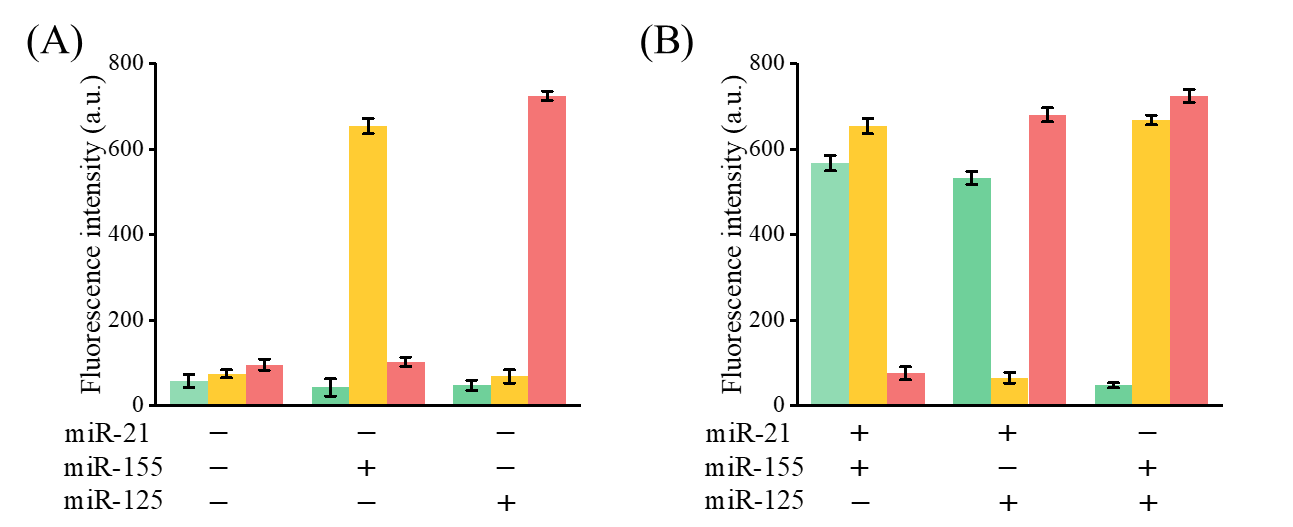


**Fig. S3** Fluorescence intensity of the EDC-CHA system with different combinations of miRNA targets. (A) Sample 1 is Blank; Sample 2 contains miRNA-155; Sample 3 contains miRNA-125. (B) Sample 1 contains miRNA-21 and miRNA-155; Sample 2 contains miRNA-21 and miRNA-125; Sample 3 contains miRNA-125 and miRNA-155. Assay conditions: 1nM T, 20 nM ternary duplex (purified at a 1:2:3 ratio), 50 nM F, 500 nM H1, 500 nM H2 at 25°C for 3 hours. Error bars, SD, n = 3.


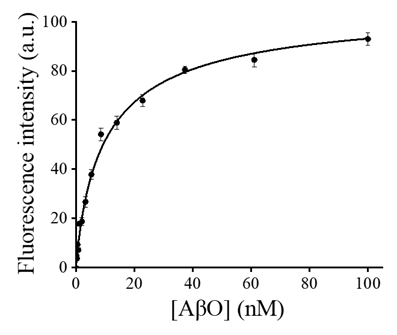


**Fig. S4** Relationship between fluorescence intensity and AβO concentration using aptamer A4. This graph illustrates how fluorescence intensity varies with increasing concentrations of AβO. The data were fitted using the LangmuirEXT1 model, yielding parameters a = 107.13, b = 0.1325, and c = 0.149. The dissociation constant was estimated as 𝐾_𝑑_≈7.54 nM. Assay conditions: 20 nM A4, varying concentration of AβO at 25°C for 1 hours. Error bars, SD, n = 3.

**RNA Extraction and miRNA Expression Analysis by** **RT-qPCR**

Total RNA was extracted from HEK-293, MCF-10A, MCF-7 and HeLa cells using a Vazyme Tissue Total RNA Isolation Kit according to the manufacturer’s instructions. The RNA concentration and integrity were quantified using a NanoDrop instrument (Bioner, South Korea), and the samples were stored at -80°C. For miRNA quantification by Real-Time PCR, 10 µl of total RNA was reverse-transcribed in a 20 µl reaction mix using the miRcute miRNA cDNA Synthesis Kit (Tiangen Biotech, Beijing, China) following the manufacturer’s recommendations. The cDNA was then used in each of the real-time PCR assays with the miRNA qPCR Detection Kit (Tiangen Biotech, Beijing, China) according to the manufacturer’s instructions. The levels of miRNAs were normalized using 5S rRNA as the reference RNA. MicroRNA expression was analyzed using the CFX96 Real-Time PCR Detection System (Bio-Rad, Hercules, CA, USA). Relative expression of the miRNA was calculated using the 2^(−∆∆ct)^ method.

Sequences of the used forward primers are as below:

miRNA-21 Forward primer: ACGTGTTAGCTTATCAGACTG

miRNA-155 Forward primer: CCGTTAATGCTAATCGTG

5S rRNA Forward primer: GCGGATAACAATTTCACACAGGA

Universal reverse primers were obtained from Tiangen Biotech (Beijing, China).


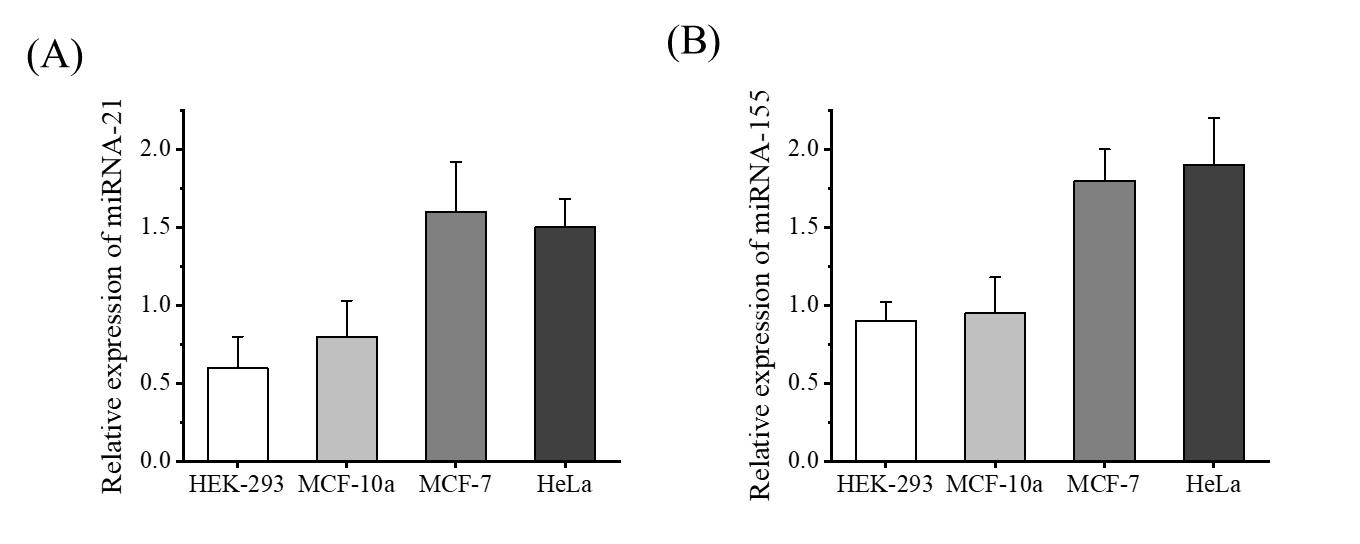


**Fig. S5** Relative expression of miR-21 (A) and miR-155 (B) in HEK-293, MCF-10A, MCF-7, and HeLa cells. RNA was extracted using Vazyme kit, quantified by NanoDrop, and stored at -80°C. cDNA was synthesized with miRcute kit (Tiangen) and analyzed by qPCR with miRNA qPCR Detection Kit (Tiangen) on CFX96 system (Bio-Rad). Expression normalized to 5S rRNA; relative levels calculated by 2^(−∆∆ct)^ method. Error bars, SD, n = 3.
